# The Treatment of Nocturnal Incontinence

**Published:** 1892-05-07

**Authors:** 


### The Practitioner's Mirror and Retrospect.

[*The Editor will be glad to receive offers of co-operation and contributions from members of the profession. All letters should be addressed to* The Editor, The Lodge, Porchester Square, London, W.]

### MEDICINE.

### THE TREATMENT OF NOCTURNAL INCONTINENCE.

It may be necessary to correct any excess of meat or starch in the diet, or extreme acidity of the urine, as well as to treat nerve diseases or local irritation such as phimosis, vulvitis, worms, &c. Next, we have to break the habit for a while, if possible, by some therapeutic means. It is worth mentioning that strips of belladonna plaster down the spine may be used successfully in place of the internal administration of the drug. Much has been said of late in praise of Rhus aromatica, or sweet Sumach, 5 to 15 drops of the extract being given twice a day. This must be continued for some months if a relapse is to be prevented. Antipyrin given in the evening, relieves some cases, but must be used with care, as it occasionally produces unpleasant results. Körner believes that incontinence is often owing to obstruction in the air passages, such as hypertrophy of the tonsils, and would direct his treatment chiefly to this. Among the various methods of electrical treatment Unverricht applies a positive electrode to the lumbar region, and moves a small negative one over the pubes. Guyon, who also uses the constant current, employs an electrode which is passed down the urethra to the membranous portion, while the kathode is placed over the perineum. Kupke prefers the Faradic current, and placed his positive pole over the last dorsal vertebra, while a small negative electrode is moved over the bladder. The sitting should not last more than five minutes. Steavenson, on the other hand, points out that by this method the sphincter vesicæ would often be missed altogether, and himself was wont to apply a negative pole against the dorsal spine, while a small positive one was placed on the perinæum. By this means he was able to use a stronger current without discomfort than if the poles had been placed in the reversed order. He too, like Guyon, preferred the constant current to the interrupted, and generally succeeded in giving relief after eight or ten sittings on consecutive days of about ten minutes each. Whatever method of treatment is employed it is important to insist on the patient emptying the bladder before going to bed, and sleeping on his side; nor must we omit to regulate any dyspepsia or constipation that may be present, nor to examine the water for evidence of kidney disease or cystitis. In the distressing cases met with in adult women where there is no reason to suspect hysteria, gradual dilation of the bladder by means of warm water is sometimes of use. The quantity poured in should be increased each time till some twenty ounces can be retained.

